# Cellular Interactions and Inflammation in the Pathogenesis of Cutaneous T-Cell Lymphoma

**DOI:** 10.3389/fcell.2020.00851

**Published:** 2020-09-04

**Authors:** Veronica Stolearenco, Martin R. J. Namini, Siri S. Hasselager, Maria Gluud, Terkild B. Buus, Andreas Willerslev-Olsen, Niels Ødum, Thorbjørn Krejsgaard

**Affiliations:** LEO Foundation Skin Immunology Research Center, Department of Immunology and Microbiology, University of Copenhagen, Copenhagen, Denmark

**Keywords:** cutaneous T-cell lymphoma, malignant T cells, fibroblasts, keratinocytes, tumor microenvironment

## Abstract

Cutaneous T-cell lymphoma (CTCL) comprises a group of lymphoproliferative diseases characterized by the accumulation of malignant T cells in chronically inflamed skin lesions. In early stages, the disease presents as skin patches or plaques covering a limited area of the skin and normally follows an indolent course. However, in a subset of patients the cutaneous lesions develop into tumors and the malignant T cells may spread to the lymphatic system, blood and internal organs with fatal consequences. Despite intensive research, the mechanisms driving disease progression remain incompletely understood. While most studies have focused on cancer cell-intrinsic oncogenesis, such as genetic and epigenetic events driving malignant transformation and disease progression, an increasing body of evidence shows that the interplay between malignant T cells and non-malignant cells plays a crucial role. Here, we outline some of the emerging mechanisms by which tumor, stromal and epidermal interactions may contribute to the progression of CTCL with particular emphasis on the crosstalk between fibroblasts, keratinocytes and malignant T cells.

## Introduction

Cutaneous T-cell lymphoma (CTCL) represents a heterogeneous group of extranodal non-Hodgkin’s lymphomas that are characterized by the accumulation of malignant T cells in chronically inflamed skin lesions. The classical clinical variants of CTCL are mycosis fungoides (MF) and Sézary syndrome (SS). Early stages of MF present as erythematous skin patches or plaques covering a limited area of the skin and closely resemble benign inflammatory skin conditions such as psoriasis and chronic eczema (Kim et al., 2005; Willemze et al., 2019). The majority of patients diagnosed with early MF experience an indolent disease course with a favorable prognosis. However, about a third of the patients progress to advanced stages which can result in a fatal outcome (Scarisbrick et al., 2014; Hristov et al., 2019). As the disease progresses, the malignant T cells accumulate, the skin lesions expand and distinctive fungus-like tumors may develop. Eventually, the malignant T cells can spread to the blood, lymph nodes, bone marrow and internal organs. With advancing clinical stages, the disease becomes increasingly aggressive, the prognosis worsens and the median life expectancy of patients with late stage CTCL drops to less than 5 years (Kim et al., 2005; Scarisbrick et al., 2014; Hristov et al., 2019; Willemze et al., 2019). SS is a leukemic form of CTCL involving generalized erythroderma, lymphadenopathy and the presence of atypical T cells with cerebriform nuclei (Sézary cells) in the peripheral blood (Kim et al., 2005; Willemze et al., 2019). It may develop *de novo* or occasionally in patients with long-term chronic MF, and is considered a late stage of CTCL due to its high aggressiveness and poor prognosis (Kim et al., 2005; Scarisbrick et al., 2014; Hristov et al., 2019; Willemze et al., 2019).

The malignant T cells in MF and SS typically exhibit the phenotype of skin-homing CD4 T cells expressing receptors such as cutaneous lymphocyte antigen (CLA) and CC chemokine receptor 4 (CCR4) (Ferenczi et al., 2002; Campbell et al., 2010; Sugaya et al., 2015). Yet, as highlighted by recent single-cell RNA sequencing studies the malignant T cells display substantial inter- and intra-patient phenotypic heterogeneity (Buus et al., 2018; Gaydosik et al., 2019). Extensive inter-patient heterogeneity is also observed at the genetic level and based on current data the disease is generally not caused by a few specific recurrent genetic aberrations (Choi et al., 2015; da Silva Almeida et al., 2015; Kiel et al., 2015; McGirt et al., 2015; Ungewickell et al., 2015; Wang et al., 2015; Woollard et al., 2016; Iyer et al., 2020; Phyo et al., 2020). Moreover, a nationwide study of Danish twins did not detect any familial aggregation of CTCL, arguing against heredity as a dominant etiologic factor (Odum et al., 2017). Somatic genetic alterations are, however, frequently observed in genes involved in certain cellular processes and signaling pathways. In particular, genes involved in epigenetic regulation, DNA damage response, cell cycle control and programmed cell death as well as in the T cell receptor (TCR), nuclear factor-kappa B (NF-κB) and Janus kinase (JAK)/signal transducer and activator of transcription (STAT) signaling pathways (Choi et al., 2015; da Silva Almeida et al., 2015; Kiel et al., 2015; McGirt et al., 2015; Ungewickell et al., 2015; Wang et al., 2015; Woollard et al., 2016; Iyer et al., 2020; Phyo et al., 2020). Importantly, extensive experimental data from cell lines, primary cells and clinical samples corroborate that dysregulation of these cellular processes and signaling pathways plays a central functional role in the pathogenesis of CTCL.

For long, it has been the general view that CTCL is a monoclonal disease with MF originating from skin-resident memory T cells and SS from mature central memory T cells (Kim et al., 2005; Campbell et al., 2010). Challenging this view, Iyer et al. (2019) recently reported the existence of multiple malignant T cell clones in both the skin and blood of MF patients with substantial variation in the clonotypes between patients and different lesions within the same patient. They further found evidence of extensive genetic intratumoral heterogeneity showing a branched phylogenetic relationship pattern (Iyer et al., 2020). Stage progression was associated with increased intratumoral heterogeneity and divergent subclonal evolution (Iyer et al., 2020). The authors proposed that MF skin lesions are formed by seeding of circulating malignant T cell clones which expand and undergo additional mutational evolution in the skin leading to the appearance of new genetically different subclones, some of which may reenter the circulation and seed other skin lesions (Iyer et al., 2020). If correct, this theory could bear significant implications for the understanding of the disease and the development of new therapeutic strategies.

The only known treatment with the potential to cure CTCL is allogenic bone marrow transplantation which is only suitable for a fraction of patients with advanced disease (Hosing et al., 2015; Johnson et al., 2019; Novelli et al., 2019). Therefore, the current therapeutic aim is primarily to control the disease, reduce symptoms and improve cosmetics while minimizing toxic effects. Early disease stages are often treated with skin-directed therapies such as topical corticosteroids and UV light therapy, whereas advanced disease usually is treated with systemic therapies (Belloni et al., 2012; Trautinger et al., 2017; Hristov et al., 2019; Trager and Geskin, 2019). However, even with proper treatment a considerable subset of CTCL patients develop or suffer from progressive disease (Belloni et al., 2012; Scarisbrick et al., 2014; Hristov et al., 2019). In view of the increased aggressiveness and poor survival rate in advanced clinical stages, it is critical to gain a better understanding of the mechanisms that drive the transition from early indolent to progressive and advanced disease.

Despite the heterogeneous nature of CTCL, consistent changes are typically observed in the lesional skin when comparing early and advanced stages. These changes appear to be facilitated by complex cellular interactions between the malignant T cells and their microenvironment and to play a central role in the progression of the disease (Miyagaki and Sugaya, 2014; Gonzalez et al., 2016; Krejsgaard et al., 2017). While most reviews have focused on the malignant T cells and their interactions with benign immune cells, we here outline some of the mechanisms by which malignant, stromal and epidermal interactions may contribute to the progression of CTCL with particular focus on emerging data highlighting the significance of the crosstalk between the malignant T cells, fibroblasts and keratinocytes.

## Th2-Bias During Disease Progression

**FIGURE 1:**
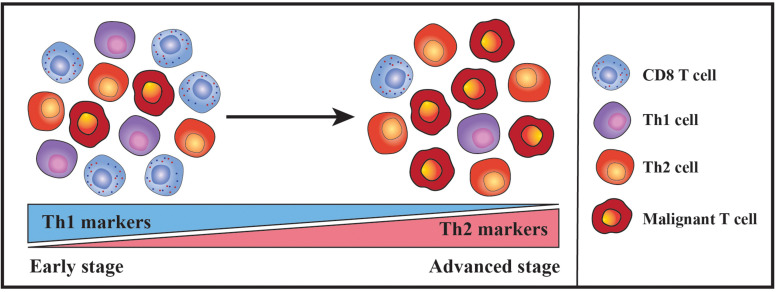
Schematic illustration portraying the shift in the nature of the inflammatory milieu during the progression of CTCL. In early disease stages, CTCL skin lesions typically contain few malignant T cells within a dense infiltrate of benign immune cells. A substantial proportion of the benign immune cells are reactive T helper 1 (Th1) cells and cytotoxic CD8 T cells and accordingly Th1-associated markers are highly expressed. However, during disease progression there is a decline in the expression of Th1-associated markers along with the numbers of infiltrating Th1 and CD8 T cells. In contrast, the malignant T cells accumulate and the levels of Th2-associated markers increase, eventually leading to a Th2-dominated inflammatory milieu in advanced disease stages.

In early stages of MF, the majority of immune cells in the lesional skin are benign and the malignant T cells only constitute a minor fraction. A substantial proportion of the benign immune cells are reactive T helper 1 (Th1) cells and cytotoxic CD8 T cells expressing interferon gamma (IFNγ) and cytotoxic molecules (Wood et al., 1994; Asadullah et al., 1997; Bagot et al., 1998; Echchakir et al., 2000; Vermeer et al., 2001; Kim et al., 2005; Hsi et al., 2015). These cells have the capacity to kill autologous malignant T cells *ex vivo* and high numbers of lesional CD8 T cells are associated with a favorable prognosis, indicating that the cellular immune reaction in early disease represents an anti-tumor response that keeps the malignant population in check (Hoppe et al., 1995; Berger et al., 1996; Bagot et al., 1998; Vermeer et al., 2001; Abeni et al., 2005). However, with advancing clinical stages there is a decline in Th1-associated markers as well as in the numbers of activated Th1 and CD8 T cells, whereas the levels of Th2-associated markers including GATA-3, IL-4, IL-5 and IL-13 increase (Vowels et al., 1994; Hoppe et al., 1995; Vermeer et al., 2001; Papadavid et al., 2003; Hahtola et al., 2006; Johnson et al., 2014; Litvinov et al., 2014; Geskin et al., 2015; Hsi et al., 2015). The shift from a Th1- to a Th2-biased tumor microenvironment (Figure 1) is thought to play a critical role in the transition from indolent to progressive disease by impairing cellular anti-tumor responses whilst fostering the proliferation of malignant T cells. Indeed, administration of Toll-like receptor (TLR) agonists boosting cellular immunity has shown clinical efficacy, and treatment with IL-12 and IFNγ can induce regression of CTCL lesions which is associated with increased numbers of CD8 T cells in the resolving skin (Rook et al., 1999, 2001, 2015; Suchin et al., 2002; Dummer et al., 2004; Duvic et al., 2006; Wysocka et al., 2007; Kim et al., 2010; Accart et al., 2013). Of notice, recent case reports have surprisingly described that long-term treatment with dupilumab, a neutralizing antibody targeting IL-4 receptor alpha, may exacerbate CTCL and possibly even trigger the disease in certain patients with severe atopic dermatitis (AD) (Chiba et al., 2019; Espinosa et al., 2020; Miyashiro et al., 2020; Tran et al., 2020; Umemoto et al., 2020). While substantial data currently support that the transition from a Th1- to a Th2-biased tumor microenvironment contributes to the progression of CTCL, these new findings suggest that the role of the cytokine milieu might be more complex than appreciated thus far. Further studies are, however, required to gain a better understanding of the clinical and biological effects of dupilumab in patients with CTCL.

## Cellular Interactions Enhance Malignant Stat Activation, Proliferation and Th2 Cytokine Expression

The progression of CTCL is associated with a gradual dysregulation of the JAK/STAT pathway, and evidence pinpoints this dysregulation as a driving force in mediating the shift toward a Th2-biased tumor microenvironment. Normally, STAT3, STAT5 and STAT6 become persistently activated in the malignant T cells which have been shown to fuel their expression of Th2 cytokines (Zhang et al., 1996, 2000; Nielsen et al., 2002; Sommer et al., 2004; Krejsgaard et al., 2006; Choi et al., 2015; Geskin et al., 2015; Kiel et al., 2015; Woollard et al., 2016; Gaydosik et al., 2020). Furthermore, activation of STAT6 upregulates the malignant expression of the Th2-associated transcription factor GATA-3 (Gaydosik et al., 2020). Whereas the expression of GATA-3 increases during disease progression, STAT4, which promotes Th1 differentiation and IFNγ expression, is often lost (Nebozhyn et al., 2006; Johnson et al., 2014; Litvinov et al., 2014; Hsi et al., 2015). Accordingly, malignant T cells isolated from patients with advanced leukemic disease typically express Th2 cytokines such as IL-4 and IL-13 but are negative for IFNγ (Guenova et al., 2013).

While it remains unclear what initially triggers an increase in the lesional levels of Th2 cytokines, evidence suggests that this may ignite a positive feedback loop between the malignant, stromal and epidermal cells that further enhances the malignant activation of STAT proteins and expression of Th2 cytokines. For example, it has been shown that IL-4 and IL-13 stimulate dermal fibroblasts from CTCL patients to secrete increased levels of the extracellular matrix protein periostin (Takahashi et al., 2016). Periostin is known to induce expression of thymic stromal lymphopoietin (TSLP) from keratinocytes and both periostin and TSLP are elevated in CTCL skin lesions and serum when compared with skin and serum from healthy control subjects (Miyagaki et al., 2009; Tuzova et al., 2015; Takahashi et al., 2016). The lesional levels of IL-4, periostin and TSLP correlate, indicating a scenario where Th2 cytokines stimulate dermal fibroblasts to secrete periostin which induces expression of TSLP from the epidermal keratinocytes. Completing the circle, TSLP has in turn been shown to activate STAT5 in malignant CTCL cells thereby promoting both their proliferation and production of IL-4 and IL-13 (Takahashi et al., 2016). Notably, STAT5 can also downregulate the malignant expression of STAT4 and the chromatin organizer and transcription factor SATB1 through induction of microRNA-155 (miR-155) (Litvinov et al., 2014; Fredholm et al., 2018; Herrera et al., 2020). As SATB1 represses the expression of IL-5 and IL-9 in the malignant T cells, activation of STAT5 may in this manner indirectly promote the expression of these cytokines while concurrently suppressing IFNγ (Fredholm et al., 2018; Herrera et al., 2020).

**FIGURE 2:**
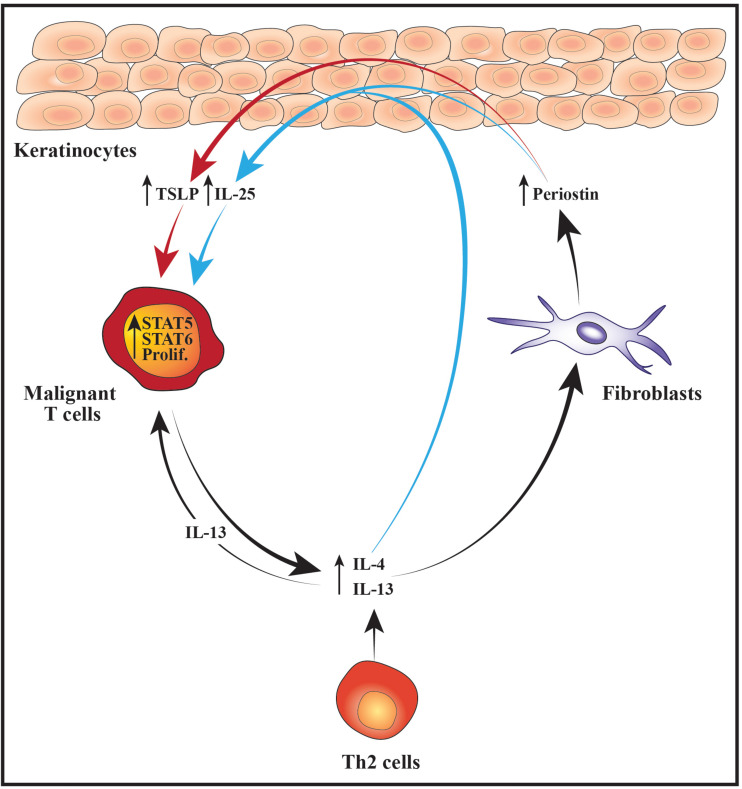
Schematic illustration of cellular interactions that may fuel the malignant STAT activity, proliferation and Th2 cytokine expression. Increased expression of IL-4 and IL-13 from malignant T cells and/or benign Th2 cells stimulates dermal fibroblasts to produce higher levels of periostin which subsequently promotes the secretion of TSLP from the epidermal keratinocytes. In parallel, IL-4, IL-13 and periostin cooperatively stimulate the keratinocytes to express IL-25. The elevated levels of TSLP and IL-25 boost the activity of STAT5 and STAT6 in the malignant T cells which fuel their proliferation (prolif.) and production of Th2 cytokines such as IL-4 and IL-13. In turn, IL-13 further augments the malignant proliferation in an autocrine and paracrine manner. Moreover, the increased expression of Th2 cytokines leads to enhanced secretion of periostin, TSLP and IL-25 thus giving rise to a positive feedback loop nurturing the malignant T cells.

Periostin, IL-4 and IL-13 have in addition been reported to stimulate primary human keratinocytes to express increased levels of the cytokine IL-25, which promotes Th2 immunity and cytokine production (Xu and Dong, 2017; Nakajima et al., 2018). Accordingly, the expression of IL-25 is increased in the epidermal keratinocytes in advanced CTCL skin lesions and enhances the expression of IL-13 in IL-25 receptor-positive malignant T cells via activation of STAT6 (Nakajima et al., 2018). Extending these data, Geskin et al. (2015) demonstrated that malignant T cells in the skin and blood of CTCL patients express receptors for IL-13. The authors further provided evidence that IL-13 promotes the proliferation of malignant T cells isolated from the blood of leukemic CTCL patients, suggesting that IL-25, TSLP and the general skewing toward a Th2-polarized inflammatory environment may fuel the malignant expansion indirectly by augmenting the levels of IL-13 (Geskin et al., 2015). Taken together, these findings illustrate how an initial increase in Th2 cytokines may elicit a complex loop of continuous signaling between fibroblasts, keratinocytes and malignant T cells that sustains and enhances the activation of STAT proteins, the proliferation and the Th2 cytokine expression of the malignant T cells (Figure 2).

## Changes in the Malignant Cytokine Expression Foster a Th2-Biased Inflammatory Environment

Accumulating evidence indicates that the skewing of the malignant cytokine expression toward a Th2 profile fosters the development of a Th2-biased inflammatory environment – both via direct effects on the benign immune cells and indirectly by modulating the chemokine expression pattern in the tumor microenvironment (Miyagaki and Sugaya, 2014; Gonzalez et al., 2016; Krejsgaard et al., 2017). The capacity of the malignant T cells to directly modulate the inflammatory response of benign immune cells was elegantly demonstrated by Guenova et al. (2013). The authors found that malignant and benign T cells from leukemic CTCL patients expressed lower levels of IFNγ and higher levels of IL-4 and IL-13 than T cells from healthy controls. However, when the benign and malignant T cells were cultured separately, the benign T cells expressed enhanced levels of IFNγ and lower levels of IL-4, whereas the expression of these cytokines remained constant in the malignant T cells (Guenova et al., 2013). Peripheral blood mononuclear cells (PBMCs) from leukemic CTCL patients were further shown to suppress the expression of IFNγ from healthy donor PBMCs. The suppressive effect was completely abrogated by neutralizing antibodies against IL-4 and IL-13, suggesting that the malignant T cells directly repress benign Th1 responses via their expression of Th2 cytokines (Guenova et al., 2013). Supporting that the malignant T cells play a key role in suppressing Th1 responses in CTCL patients, different treatment modalities which reduced the numbers of malignant T cells through distinct mechanisms of action were invariably associated with enhanced Th1 and reduced Th2 responses (Guenova et al., 2013). Of notice, the malignant T cells may also inhibit anti-tumor immunity by inducing apoptosis in the benign T cells. It was, for example, recently reported that the malignant T cells secrete galectin-9 and that increased expression of galectin-9 in CTCL skin lesions is associated with a reduced infiltration of CD8 T cells while high serum levels are correlated with disease severity markers (Nakajima et al., 2019). Likewise, the malignant T cells frequently express Fas ligand (FasL) and have been shown to trigger FasL-dependent T cell apoptosis *in vitro* (Ni et al., 2001, 2005). CD8 T cells were reported to be inversely distributed with FasL-expressing malignant T cells in CTCL skin lesions, implying that the malignant T cells might use FasL to eliminate tumor-reactive CD8 T cells (Ni et al., 2001).

**FIGURE 3:**
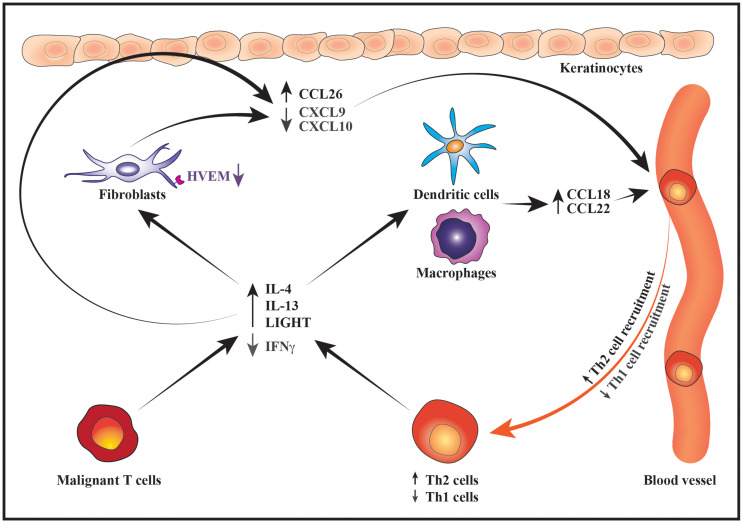
Schematic illustration of how changes in the malignant cytokine secretion alter the chemokine expression pattern to promote a Th2-biased inflammatory microenvironment. During disease progression, the malignant T cells express increased levels of IL-4 and IL-13 whereas their expression of IFNγ decreases. Lower production of IFNγ by malignant T cells together with downregulation of HVEM receptor on fibroblasts leads to suppressed expression of the Th1-recruting chemokines CXCL9 and CXCL10 by fibroblasts and keratinocytes resulting in reduced recruitment of Th1 cells into the tumor microenvironment. In contrast, increased secretion of IL-4 and IL-13 stimulates the production of Th2-recruting chemokines such as CCL26 by keratinocytes and fibroblasts as well as CCL18 and CCL22 by macrophages and DCs. In turn, the recruitment of more Th2 cells to the lesional skin contributes to the increasing levels of Th2 cytokines thereby sustaining the development of a Th2-dominated tumor microenvironment.

The malignant T cells may not only foster a Th2-dominated tumor microenvironment through direct effects on the benign immune cells but also indirectly by modulating the chemokine expression pattern of fibroblasts, macrophages, dendritic cells (DCs) and keratinocytes. In early disease the keratinocytes and dermal fibroblasts express high levels of chemokines such as CXCL9 and CXCL10 which preferentially attract CD8 T cells and Th1 cells. The expression of these Th1-associated chemokines is, however, strongly reduced in advanced disease stages, whereas the expression of chemokines such as CCL17, CCL18, CCL22 and CCL26 that preferentially attract Th2 cells is increased (Sarris et al., 1995; Tensen et al., 1998; Kakinuma et al., 2003; Bromley et al., 2008; Miyagaki et al., 2010, 2012b, 2013; Tuzova et al., 2015). Providing a putative explanation for the decreased expression of CXCL9 and CXCL10 in advanced disease, Miyagaki et al. (2012b) demonstrated that IFNγ induces secretion of these chemokines from dermal fibroblasts. IFNγ-induced synthesis of CXCL9 and CXCL10 was strongly potentiated by the TNF superfamily member LIGHT. Although LIGHT was expressed both in early and advanced disease stages, the expression of its receptor, HVEM, was decreased on fibroblasts in advanced disease, potentially contributing to their reduced expression of CXCL9 and CXCL10. In contrast to IFNγ, IL-4 has been shown to stimulate dermal fibroblasts from CTCL patients to express high levels of the Th2-recruiting chemokine CCL26 *in vitro* and, accordingly, the expression levels of IL-4 and CCL26 were found to correlate in the lesional skin (Miyagaki et al., 2010). IL-4 and IL-13 are correspondingly potent inducers of CCL26 expression in human keratinocytes, whereas IFNγ stimulates keratinocytes to secrete CXCL9 and CXCL10 (Giustizieri et al., 2001; Ohta et al., 2008; Gaspar et al., 2013). The increased expression of Th2 cytokines combined with the decreased secretion of IFNγ and HVEM may therefore, at least partially, explain the observed changes in the expression pattern of CXCL9, CXCL10 and CCL26 during disease progression. In addition, Th2 cytokines are known to stimulate the secretion of CCL18 and CCL22 from antigen-presenting cells (Vulcano et al., 2001; van Lieshout et al., 2006; Tsicopoulos et al., 2013; Furudate et al., 2016). These chemokines are indeed expressed by macrophages and DCs in CTCL skin lesions and the levels of CCL18 correlate with those of IL-4 (Gunther et al., 2011; Miyagaki et al., 2013; Tanita et al., 2019; Vieyra-Garcia et al., 2019). A recent study provided evidence suggesting that lesional c-Kit^+^ DCs recruit benign Th2 cells via secretion of CCL18 which leads to formation of an inflammatory synapse between the DCs, benign Th2 cells and the malignant T cells (Vieyra-Garcia et al., 2019). In this synapse, the benign Th2 cells are activated by OX40/OX40L cell interactions with the DCs and by CD40/CD40L interactions with both DCs and malignant T cells resulting in increased skin inflammation (Vieyra-Garcia et al., 2019). Collectively, these data illustrate how changes in the malignant expression of Th1 and Th2 cytokines may modulate the chemokine expression of benign immune cells, fibroblasts and keratinocytes to favor skin trafficking and activation of Th2 cells (Figure 3). By adding to the increasing levels of Th2 cytokines, this fuels a vicious cycle that reinforces the development of a Th2-biased inflammatory tumor microenvironment (Figure 3).

## The Malignant T Cells Induce Angiogenesis and Lymphangiogensis

Angiogenesis and lymphangiogenesis are critical processes in tumor growth and metastasis (Stacker et al., 2014; De Palma et al., 2017). Accordingly, biopsies from the lesional skin of CTCL patients exhibit a stage-dependent increase in microvessel density and the lymphatic marker podoplanin (Vacca et al., 1997; Mazur et al., 2004; Kawaguchi et al., 2014; Jankowska-Konsur et al., 2016, 2017). High expression of podoplanin is associated with shorter overall survival in CTCL patients, and the expression levels of vascular and proliferative markers correlate *in situ* (Jankowska-Konsur et al., 2016, 2017). Moreover, high expression of endothelial and lymphatic markers is associated with nodal involvement, altogether indicating that angiogenesis and lymphangiogenesis may contribute to the expansion and dissemination of the malignant T cells (Jankowska-Konsur et al., 2016, 2017).

The increase in podoplanin-positive lymphatic vessels during the clinical progression of CTCL strongly correlates with the expression levels of vascular endothelial growth factor C (VEGF-C) which is a key stimulator of lymphangiogenesis (Stacker et al., 2014; Jankowska-Konsur et al., 2017). While neoplastic T cells stain positive for VEGF-C in CTCL skin lesions, malignant CTCL cells were reported not to produce VEGF-C *in vitro* (Pedersen et al., 2013). However, when the malignant T cells were inoculated into the skin of immunodeficient mice a proportion of them displayed clear expression of VEGF-C during tumor formation *in vivo* but this expression was not retained *ex vivo* (Pedersen et al., 2013). VEGF-C-positive malignant T cells were predominantly observed in close proximity to stromal cells within the tumor microenvironment. Interestingly, *in vitro* co-culture of malignant CTCL cell lines with skin fibroblasts enhanced the secretion of VEGF-C by the latter, jointly suggesting that the tumor and stromal cells may engage in a cross-talk that promotes synthesis of VEGF-C which, in turn, stimulates lymphangiogenesis (Pedersen et al., 2013).

In line with the growing microvessel density, a variety of angiogenic factors are increased in the lesional skin of CTCL patients when compared with normal skin or skin from patients with benign inflammatory skin conditions (Krejsgaard et al., 2006; Miyagaki et al., 2012a, 2017; Kawaguchi et al., 2014; Lauenborg et al., 2015; Sakamoto et al., 2018; Suzuki et al., 2020). Although some of these are expressed by stromal cells, the majority mainly appear to be produced by the malignant T cells. For instance, atypical T cells in CTCL lesions stain positive for the highly angiogenic protein VEGF-A, and malignant CTCL cell lines produce VEGF-A via a JAK- and c-Jun N-terminal kinase (JNK)-dependent mechanism *in vitro* (Krejsgaard et al., 2006; Miyagaki et al., 2017; Sakamoto et al., 2018). Besides being a potent stimulator of angiogenesis, VEGF-A also has the capacity to enhance the expression of TSLP in keratinocytes and, accordingly, the serum concentrations of VEGF-A correlate with the lesional expression of TSLP in erythrodermic CTCL patients (Sakamoto et al., 2018). VEGF-A may thus both contribute to the pathogenesis of CTCL by promoting angiogenesis, and by stimulating TSLP production by epidermal keratinocytes. Alongside VEGF-A, the malignant T cells also express lymphotoxin α (LTα) and its cognate receptor, tumor necrosis factor receptor (TNFR)2 (Lauenborg et al., 2015). It was demonstrated that LTα can function in an autocrine manner to induce malignant secretion of IL-6 which together with LTα and VEGF-A stimulate endothelial sprouting and tube formation (Lauenborg et al., 2015). The malignant T cells have additionally been reported to express a number of other pro-angiogenic factors such as IL-17F, angiopoietin-2 (Ang-2), placental growth factor (PlGF) and YKL-40 (Krejsgaard et al., 2013; Kawaguchi et al., 2014; Lauenborg et al., 2017; Miyagaki et al., 2017; Suzuki et al., 2020). The secretion of IL-17F by malignant T cells has been shown to stimulate angiogenesis *in vitro*, and the numbers of Ang-2-positive cells correlate with the numbers of blood vessels in erythrodermic CTCL lesions (Kawaguchi et al., 2014; Lauenborg et al., 2017). Furthermore, PlGF and YKL-40 were demonstrated to promote blood vessel formation and lymphoma growth in murine xenograft models (Miyagaki et al., 2017; Suzuki et al., 2020). In conjunction with angiogenic factors, the malignant T cells express matrix metalloproteinases (MMP) such as MMP2 and MMP9 which may facilitate the angiogenic process and spread of the malignant T cells (Vacca et al., 1997; Rasheed et al., 2010). MMPs were also found to be strongly expressed by stromal cells in the vicinity of the malignant T cells, suggesting that malignant T cells secrete factors that stimulate the expression of MMPs by the surrounding stroma (Vacca et al., 1997; Rasheed et al., 2010). Collectively, these data highlight how the malignant T cells may facilitate their own growth and dissemination through the production of factors that directly or indirectly stimulate blood and lymph vessel formation in the tumor stroma.

## The Malignant T Cells May Induce Changes in the Epidermal Architecture

In addition to the increased microvessel density, the lesional skin of CTCL patients often exhibits changes in the epidermal architecture and an impaired barrier function (Suga et al., 2014; Fatima et al., 2020). Accordingly, Suga et al. (2014) found that keratinocytes in CTCL skin lesions express lower levels of skin barrier proteins than keratinocytes in healthy skin. This was more prominent in advanced disease and the levels of skin barrier proteins correlated inversely with the expression of IL-4, CCL17 and CCL18. Using an organotypic skin model of CTCL, Thode et al. (2015) provided evidence that the malignant T cells secrete factors which affect the behavior of keratinocytes leading to increased proliferation, disorganized stratification and decreased resistance to mechanical stress. The malignant T cells have also been shown to produce IL-31, which together with IL-4, IL-5 and IL-13, is believed to stimulate the sensation of pruritus in CTCL patients (Ohmatsu et al., 2012; Singer et al., 2013; Cedeno-Laurent et al., 2015; Nattkemper et al., 2016; Lewis et al., 2018). At such, the malignant T cells may facilitate mechanical disruption of the skin integrity indirectly by producing factors that elicit scratching. Yet, as many of the cutaneous features of CTCL resemble those of chronic inflammatory skin diseases it is likely that interactions between the benign immune cells and keratinocytes also contribute significantly to the changes in the epidermal architecture (Ralfkiaer et al., 2011; Willemze et al., 2019). Indeed, Miyagaki et al. (2011) reported that benign T cells in CTCL produce IL-22 which may promote STAT3 activation, CCL20 expression and epidermal hyperplasia. In general, this area of investigation remains poorly explored and further studies are needed to gain a better understanding of how the interplay between malignant T cells, benign immune cells and keratinocytes affects the epidermis.

## Interactions Between Bacterial Toxins and Malignant T Cells

Considering the compromised cellular immunity and weakened skin barrier, it is not surprising that CTCL patients exhibit increased susceptibility to cutaneous bacterial infections. The lesional skin is, in particular, frequently colonized by enterotoxin-producing *Staphylococcus aureus* (*S. aureus)* which constitutes a major cause of morbidity and mortality (Axelrod et al., 1992; Tokura et al., 1995; Jackow et al., 1997; Nguyen et al., 2008; Talpur et al., 2008; Mirvish et al., 2011; Willerslev-Olsen et al., 2013; Lebas et al., 2017; Blaizot et al., 2018; Scarisbrick, 2018). A series of *in vitro* studies have uncovered that staphylococcal enterotoxins can trigger a complex crosstalk between the malignant and non-malignant T cells. This cross-talk leads to increased proliferation, cytokine production, IL-2 receptor alpha chain (IL2Rα) expression and STAT3 activation in the malignant T cells, suggesting that staphylococcal enterotoxins may modulate the inflammatory environment and fuel disease progression (Woetmann et al., 2007; Krejsgaard et al., 2014; Willerslev-Olsen et al., 2016, 2020; Lindahl et al., 2019). *S. aureus* also produces other factors that may play a pathogenic role in CTCL. It was for example shown that *S. aureus* isolates from CTCL skin lesions express the pore forming toxin, alpha-toxin, and that the malignant T cells in many patients are considerably more resistant to alpha-toxin-induced cell death than non-malignant CD4 and CD8 T cells (Blümel et al., 2019, 2020; Lindahl et al., 2019). It is thus possible that alpha-toxin impedes CD8 T cell-mediated anti-tumor responses and tilts the balance between the malignant and non-malignant CD4 T cells. Supporting that toxinogenic *S. aureus* infections exacerbate the disease activity, antibiotic treatment leading to successful eradication of *S. aureus* is associated with significant clinical improvement in most patients with advanced CTCL (Tokura et al., 1995; Jackow et al., 1997; Talpur et al., 2008; Willerslev-Olsen et al., 2013; Lindahl et al., 2019). A recent study further demonstrated that patients exhibiting clinical improvement after transient antibiotic therapy, display diminished STAT3 signaling, IL-2Rα expression and cell proliferation in the lesional skin (Lindahl et al., 2019). Antibiotic treatment not only reduced the disease activity at the clinical and histological level but also resulted in a significant decrease in the fraction of malignant T cells in the lesional skin in the majority of patients (Lindahl et al., 2019). These findings indicate that there may be a mutual sustenance between the malignant T cells and toxin-producing *S. aureus* where the former compromise the local immunity and the bacteria, in turn, mitigate anti-tumor responses while concurrently fueling the expansion of malignant T cells.

## Conclusion

Accumulating evidence suggests that malignant, stromal and epidermal interactions play a central role in the pathogenesis of CTCL. In this review, we have outlined some of the emerging mechanisms by which these interactions may contribute to the progression of the disease. As highlighted, complex signaling networks between fibroblasts, keratinocytes and the tumor cells may fuel diverse pathological processes including the malignant activation of STAT proteins, the development of a Th2 dominated inflammatory microenvironment, neovascularization of the tumor tissue and changes in the skin architecture. These processes can through different pathways facilitate the malignant proliferation and dissemination while impeding anti-tumor responses. Despite recent progress, the interactions between tumor, stromal and epidermal cells in CTCL remain poorly characterized and additional studies are warranted to substantiate and further illuminate their role in the pathogenesis of the disease. We believe that research into this field of investigation may pave the way for novel therapeutic strategies that can be of clinical benefit for patients with progressive or advanced CTCL.

## Author Contributions

VS, MN, SH, MG, TB, AW-O, NØ, and TK wrote the manuscript. VS and TK made the figures. All authors contributed to the article and approved the submitted version.

## Conflict of Interest

The authors declare that the research was conducted in the absence of any commercial or financial relationships that could be construed as a potential conflict of interest.
